# Gamma passing rates of daily EPID transit images correlate to PTV coverage for breast cancer IMRT treatment plans

**DOI:** 10.1002/acm2.13913

**Published:** 2023-01-26

**Authors:** David Sánchez‐Artuñedo, Victoria Reyes López, Raquel Granado Carrasco, Mercè Beltran‐Vilagrasa, Maria Amor Duch‐Guillen, Marcelino Hermida‐López

**Affiliations:** ^1^ Servei de Física i Protecció Radiològica Hospital Universitari Vall d'Hebron Vall d'Hebron Barcelona Hospital Campus Barcelona Spain; ^2^ Servei d'Oncologia Radioteràpica Hospital Universitari Vall d'Hebron Vall d'Hebron Barcelona Hospital Campus Barcelona Spain; ^3^ Institut de Tècniques Energètiques Universitat Politècnica de Catalunya. Barcelona Spain

**Keywords:** breast, Halcyon, transit dosimetry

## Abstract

**Purpose:**

The use of the transit image obtained with the electronic portal‐imaging device (EPID) is becoming an extended method to perform in‐vivo dosimetry. The transit images acquired during each fraction can be compared with a predicted image, if available, or with a baseline image, usually the obtained in the first fraction. This work aims to study the dosimetric impact of the failing fractions and to evaluate the appropriateness of using a baseline image in breast plans.

**Material and methods:**

Twenty breast patients treated in a Halcyon were retrospectively selected. For each patient and fraction, the treatment plan was calculated over the daily CBCT image. For each fraction, the differences respect to the treatment plan values of OARs and PTV dosimetric parameters were analyzed: ΔD_mean_, ΔD95%, ΔD98%, ΔD2%, ΔV36Gy, ΔV38.5Gy, and ΔV43.5Gy.

Daily fractions were ranked according to the differences found in the dosimetric parameters between the treatment plan and the daily CBCT to establish the best fraction. The daily transit images acquired in every fraction were compared to the first fraction using the global gamma index with the Portal Dosimetry tool. The comparison was repeated using the best fraction image as a baseline.

We assessed the correlation of the dosimetric differences obtained from the CBCT images‐based treatment plans with the gamma index passing rates obtained using first fraction and best fraction as baseline.

**Results:**

Average values of ‐11.6% [‐21.4%, ‐3.3%] and ‐3.2% [‐1.0%, ‐10.3%] for the ∆PTVD98% and ∆PTVD95% per every 10% decrease in the passing rate were found, respectively.

When using the best fraction as baseline patients were detected with failing fractions that were not detected with the first fraction as baseline.

**Conclusion:**

The gamma passing rates of daily transit images correlate with the coverage loss parameters in breast IMRT plans. Using first fraction image as baseline can lead to the non‐detectability of failing fractions.

## 1 INTRODUCTION

Several consensus protocols are available^1^, ^2^, ^3^ to guide the implementation of comprehensive quality assurance (QA) programs of clinical linear accelerators (linac). With the spread of complex treatment techniques as IMRT or VMAT, scientific societies published specific pre‐treatment QA protocols.^4^, ^5^, ^6^ As dose conformation increased^7^ with these techniques, so did the concern of a correct treatment delivery. Image‐guided radiation therapy (IGRT) was introduced to verify the correct patient positioning.^8^ However, neither IGRT nor well‐designed pre‐treatment and linac QA programs can always guarantee a correct treatment delivery. As an example, an incorrectly placed bolus on the patient skin cannot be detected neither with patient‐specific QA nor with linac QA.

In‐vivo dosimetry has proved to be a helpful tool to detect delivery‐related problems and to improve safety in radiotherapy treatments.^9^ Initially, in‐vivo dosimetry was performed by placing point detectors at selected locations on the patient surface and measuring entrance doses during treatment delivery. However, in‐vivo dosimetry with point dosimeters is time‐consuming^10^ and the positioning uncertainty of the detector reduces its usefulness for IMRT and VMAT treatments.^11^ To overcome the limitations of in‐vivo dosimetry with point detectors, the use of transit images is becoming an extended practice.^10^, ^12^ A transit image is formed by the signal produced by radiation reaching the electronic portal‐imaging device (EPID) through the patient during irradiation of a treatment field. Transit images obtained in different treatment fractions can be compared to each other using the gamma index,^13^ with tools such as the Portal Dosimetry module included in the ARIA system (Varian Medical Systems, Palo Alto, California) or with other commercial software.

Breast cancer is one of the most frequent cancers and therefore one of the most common treatment sites in radiotherapy.^14^ In breast cancer treatments, multiple error sources can lead to a deviation between the planned and the delivered dose distributions: patient rotations, patient shifts, changes in breast shape, etc.^15^ This wide variety of error sources highlights the importance of in‐vivo dosimetry in the treatment of breast cancer patients. Early works on breast transit dosimetry^16^ used EPID images to manually calculate the delivered dose at a specific point in the planning CT. The delivered dose obtained from the transit images was then compared to the planning point dose value. Subsequently, commercial software such as Dosimetry Check (Math Resolutions LLC) was developed to automatically calculate dose at a reference point. Nowadays, several commercial systems are available that implement automated in‐vivo dosimetry based on EPID images.^10^ Some examples are Adaptivo (Standard Imaging, Wisconsin, USA), Dosimetry Check (currently distributed by LAP, Texas, USA) and the PerFraction software (Sun Nuclear Corporation, Melbourne, USA).

PerFraction can analyze EPID images and treatment log files for every treatment fraction. Daily EPID images can be used in two ways: (1) compared to a baseline image acquired in previous fractions, or (2) compared to a predicted transit image, which is calculated by PerFraction assuming that the treatment is delivered as planned. In both options, images can be compared using the gamma index with different criteria. Ideally option two would be preferable, as it allows for true in‐vivo dosimetry. Unfortunately, transit dosimetry is not available for the Halcyon linac at the present time with PerFraction. Therefore, Halcyon users who want to perform transit dosimetry can use the daily EPID images and compare them using methodology 1.

Despite the literature, it is not clear that differences in the transit dosimetry correlate to a decrease in the PTV's coverage dose. Moreover, it is not clear the validity of using baseline images as a surrogate of a real in‐vivo dosimetry using predicted dose images. The aim of this work is twofold: (1) to study the correlation between PTV parameters and gamma analysis of the transit images and (2) to evaluate the appropriateness of using a baseline image for transit dosimetry in breast treatment plans.

## 2 METHODS

Twenty randomly selected breast cancer patients without lymph node involvement were included in this retrospective study. Twelve left‐sided and eight right‐sided breasts treatments were analyzed. All these patients followed a hypofractionated scheme of 15 fractions with a prescribed dose of 40.5 Gy to the breast and 48 Gy to the simultaneous integrated boost.

Patients were treated in a Halcyon 2.0 system (Varian Medical Systems). Target volumes and organs at risk were contoured according to RTOG guidelines.^17^ Patients were immobilized using BreastSTEP (Elekta) inclined plane. Simulation and treatment were performed in free‐breathing mode.

Sliding window IMRT plans were calculated using the Eclipse treatment planning system (Varian Medical Systems) using the analytical anisotropic algorithm (AAA) v. 15.6.4. All plans included 6 MV flattening‐filter‐free fields and were calculated using a 2.5 mm grid. Depending on the complexity of the treatment volume, four to six beams were used in the plans. First and last beam correspond to the inner and outer tangential beam, respectively. In the tangential fields, photon fluence was extended outside the body contour with the skin flash tool. The rest of the fields were placed between these two at variable gantry angles depending on the patient geometry.

Treatment setup was verified with kV‐CBCT, as the orthogonal 2D kV‐kV modality is not available in the Halcyon 2.0. In this linac, daily setup image and online correction are mandatory, by design, prior to treatment. If the translational differences were below 1 cm, a 3D couch correction was applied (as Halcyon does not allow rotation correction). Otherwise, the patient was repositioned on the linac and new images were acquired. If the new matching values were still out of tolerance, the oncologist decided to treat applying matching values or to resimulate the patient.

We used the daily CBCT acquired prior treatment to study the changes in the dosimetric parameters. The treatment plan and the contoured structures were copied over the daily CBCT for each fraction based on the registration performed online in the treatment unit. For each patient, the treatment plans were recalculated on the daily CBCT. The CBCT‐based plans were recalculated with the same monitor units per field as the original plan.

The Hounsfield Units (HU) of CBCT slices depend on the image parameters and on the radiation scattered by the patient.^18^ This patient‐specific HU difference implies a difference in the electron density and, therefore, in the calculated dose.^19^, ^20^ To remove this patient‐specific effect on the calculated dose between the CT and the CBCT, the density of water was assigned to the PTV and to the skin (defined as a 5 mm inner wall margin of the external contour of the patient) in the daily CBCT. In addition, the ipsilateral lung structure was overridden with ‐700 HU (Eclipse lung HU value). The same density override was made in a copy of the planning CT (pCT) and the original plan was also recalculated with the same monitor units. In this way, no bias was added to the calculated dose.

For each plan, the following dosimetric parameters of the breast PTV (PTV breast minus a 3 mm margin for the boost PTV) were extracted from the dose distributions using the Eclipse Scripting Application Programming Interface: D_mean_, D95%, D98%, D2%, V36Gy, V38.5Gy, and V43.5Gy. Regarding the OARs we studied D_mean_, V5Gy, V10Gy, V17Gy, and V20Gy for the lung and D_mean_ and D_max_ for the heart. For each treatment fraction, we calculated the difference of each dosimetric parameter with respect to the corresponding values in the pCT: ΔD_mean_, ΔD95%, ΔD98%, ΔD2%, ΔV36Gy, ΔV38.5Gy, and ΔV43.5Gy for the PTV and we used the same notation for the OARs.

Moreover, daily fractions were ranked according to the differences found in the dosimetric parameters between the pCT and the daily CBCT. Rank 1 represented the administered fraction most similar to the treatment plan and rank 15 represented the fraction with the greatest difference. The objective was to evaluate if using the first fraction image as baseline was the optimum choice. Once this ranking was established, the “best fraction” was obtained as the one with the smallest differences respect to the pCT.

Transit images were acquired with the Digital Megavoltage Images (DMI) panel. The DMI panel is placed at a fixed source‐imager distance of 154 cm. Size of the detector is 43 × 43 cm^2^, and images are represented at a 1280 × 1280 pixel matrix. Transit images are acquired using the dosimetry mode. Dosimetry mode is calibrated so that a delivered field of 100 monitor units with a field size of 10 × 10 cm^2^ produces a signal of one calibration unit (CU) at the central axis. In reference conditions, the measured reproducibility is less than 0.5%. We used the inner tangential field transit images acquired during every treatment fraction to study the correlation of the gamma analysis of the transit images with the previously defined parameters. We chose this field because the interpretation of the obtained images is intuitive as they show a tangential projection of the PTV. Initially, we established the first fraction transit image as a baseline for comparison. The daily transit images acquired in the rest of the treatment fractions were compared to the baseline using the global gamma index with the Portal Dosimetry tool. Then, we repeated the process establishing the best fraction image as a baseline for comparison. As a preliminary part of the study, the gamma criterion used to analyze the transit images was determined. For a given gamma criterion the passing rate is defined as the percentage of points studied with gamma value < 1. When the passing rate obtained using the selected gamma criterion is below the passing rate set as tolerance, the fraction was considered a “failing fraction.” The objective was to establish a gamma criterion that led to approximately 10% of failing fractions with a passing rate of 90%. Four criteria were studied: γ(2%/2 mm, threshold (TH) = 10%), γ(3%/3 mm, TH = 10%), γ(5%/3 mm, TH = 10%), and γ(7%/5 mm, TH = 20%). The percentages of failing fractions were 54.3%, 24.6%, 13.9%, and 4.3% for each gamma criteria, respectively. We set the gamma criteria in this study at γ(5%/3 mm, TH = 10%) since we obtained the closest number of failing fractions to 10%.

We studied the linear correlation between the passing rate obtained depending on the baseline image used and the change in the dosimetric parameters. The goodness of the fit was assessed using the Pearson correlation coefficient (*r*). Correlation was considered when *r* > 0.40. In this case correlations were categorized into moderate correlation (0.40 < *r* < 0.70) and strong correlation (*r* ≥ 0.70).^21^ We evaluated the change in these parameters for each 10% loss of passing rate from the slope of the linear fit of the data to determine the dosimetric impact. As an example, this parameter implies that if the ∆D95% change for every 10% passing rate is ‐5%, in a fraction with a passing rate of 90%, there will be a change in the D95% with respect to the planned value of ‐5%. If the passing rate is 80% a change of ‐10%, and so on.

In the failing fractions, we reviewed the CBCT image acquired in Halcyon prior to treatment to establish a possible cause of the failure. The causes were categorized into suboptimal positioning, changes in patient anatomy, or both. We studied whether the different causes of error produced similar areas with gamma values > 1 in the transit images. If that was the case, the cause of failure could be established by observing the areas where the gamma criteria were not met in the transit image.

## 3 RESULTS

**FIGURE 1:**
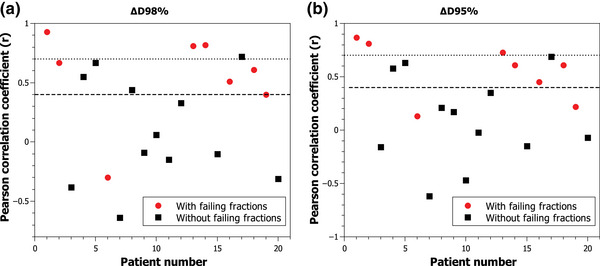
Correlation between (a) ∆D98% and (b) ∆D95% and passing rates with γ(5%/3 mm, TH = 10%) criteria using the first fraction as baseline. The dashed line represents the area of moderate correlation and the dotted line represents the area of strong correlation

A total of 300 transit images of the inner tangential fields were obtained from the 20 patients treated in the Halcyon unit. Figure 1 shows the correlation between (a) ∆D98% and (b) ∆D95% and the passing rates obtained with the γ(5%/3 mm, TH = 10%) criteria using the first fraction as baseline. Of eight patients with failing fractions, three showed a strong correlation and three a moderate correlation. In addition, three patients without failing fractions presented a moderate correlation and for ∆D95% one patient presented a strong correlation. For ∆D98%, patient number 6 in Figure 1a) showed a negative correlation and had failing fractions.

In only five patients the first fraction was the most similar fraction to the treatment plan. Moreover, in 11 patients the first fraction ranked (according to the differences found in the dosimetric parameters between the pCT and the daily CBCT) in the top five fractions, in six patients ranked between the 6^th^ and the 10^th^ and in three patients ranked in the bottom five fractions. The best fraction occurred in 10 patients during the first five fractions, in six patients during the 6th to 10th fraction and in four patients during the last five fractions.

**FIGURE 2:**
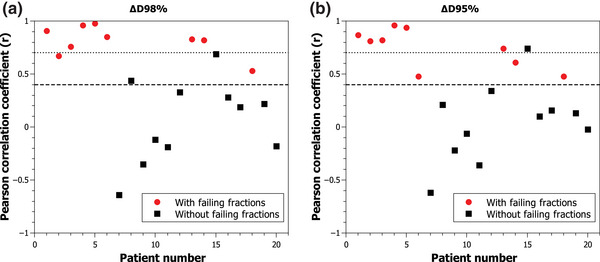
Correlation between (a) ∆D98% and (b) ∆D95% and passing rate with γ(5%/3 mm, TH = 10%) criteria using best fraction as baseline. The dashed line represents the area of moderate correlation and the dotted line represents the area of strong correlation

Figure 2 shows the correlation between (a) ∆D98% and (b) ∆D95% and the passing rates obtained with the γ(5%/3 mm, TH = 10%) criteria using the best fraction as baseline. For ∆D98%, out of nine patients with failing fractions, seven presented a strong correlation and two presented a moderate correlation. Similar results were obtained for ∆D95%, out of nine patients with failing fractions, six presented a strong correlation and three presented a moderate correlation. Only one patient without failing fractions presented a strong correlation. New failing fractions were identified for patients 3, 4, and 5 (Figure 2) that had not been detected using first fraction as baseline. The parameters with the best correlations are those related to PTV coverage. Correlation for the ΔD_mean_ was obtained in six patients over the nine with failing fractions. Seven patients presented correlations for the ΔV36Gy and eight for the ΔV38.5 Gy. Correlations were found in only four patients for the parameters related to maximum dose (ΔD2% and ΔV43.5 Gy).

**FIGURE 3:**
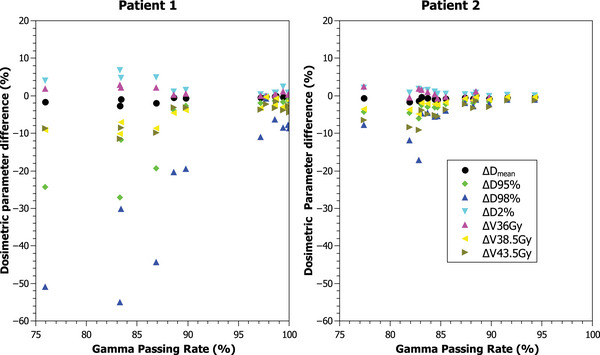
Relation between dosimetric parameters and the passing rate with γ(5%/3 mm, TH = 10%) criteria using the best fraction as baseline, for patients 1 and 2. From the slope of the linear fit to the data (not shown for clarity) we obtained the change of every dosimetric parameter per 10% loss of passing rate. Legend is only shown for one plot for clarity

Table 1 presents the change for the PTV dosimetric parameters from the nine patients with failing fractions. The bigger change was obtained in the parameters related to PTV coverage. However, the decrease in the PTV coverage varied between patients. As an example, patient 1 shows a ∆PTVD98% per every 10% passing rate loss of ‐21.4%. This implies an expected decrease in the PTVD98% of ‐21.4% if the passing rate is 90% and ‐42.8% if the passing rate is 80%. In patient 2 the ∆PTVD98% per every 10% passing rate loss was ‐7.1%. Therefore, a passing rate of 80% corresponds to an expected loss of ‐14%, less than half of the loss obtained for the patient 1. Figure 3 shows the change in the PTV dosimetric parameters for patients 1 and 2. The variability in the changes of the dosimetric parameters between two different patients can be observed. The average change, obtained from all the patients with failing fractions, of ∆PTVD98% and ∆PTVD95% per every 10% decrease in the passing rate was of ‐14% and ‐4%, respectively.

**TABLE 1:** Change in PTV dosimetric parameters per every 10% decrease in passing rate with γ(5%/3 mm, TH = 10%) criteria using best fraction as baseline

Patient	Number of fractions failing	Change in dosimetric parameter per every 10% passing rate				
		∆PTVD98%	∆PTVD95%	ΔD_mean_	ΔV36Gy	ΔV38.5 Gy
1	6	−21.4%	−10.3%	−0.7%	−3.6%	−3.1%
2	12	−7.1%	−2.8%	−0.5%	−2.6%	−4.6%
3	1	−17.8%	−4.2%	−0.9%	−3.4%	−4.2%
4	1	−18.6%	−4.4%	−1.1%	−3.7%	−6.0%
5	1	−8.7%	−2.6%		−1.8%	−2.3%
6	1	−12.3%	−1.1%		−1.9%	
13	1	−3.3%	−1.2%	0.0%		−1.6%
14	2	−10.7%	−1.0%	−0.3%		−0.2%
18	4	−4.9%	−1.0%		−1.4%	−0.5%
Average		−11.6%	−3.2%	−0.6%	−2.8%	−2.8%

*Note*: Patients without correlation for a dosimetric parameter were excluded from the analysis (marked as gray in the table). Parameters related to maximum dose in the PTV are not presented as correlations were found in only four patients.

When using first fraction as baseline similar results were obtained for all the lung dosimetric parameters than in the study of the PTV coverage. In patients with failing fraction that did not present correlation with the PTV parameters (patient 1 and 19), correlation was also not found for the lung parameters. Nevertheless, patient 2 presented failing fractions and PTV coverage correlation but no correlation in the lung parameters. A moderate correlation was found for the mean heart dose in three patients and a strong correlation in two patients. The maximum heart dose was moderately correlated in four patients.

**FIGURE 4:**
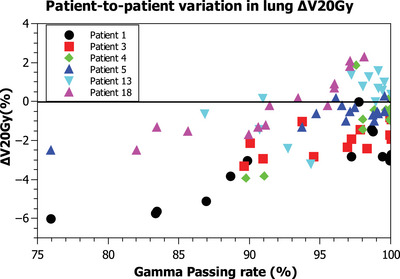
Relation between ipsilateral lung ΔV20Gy and the passing rate with γ(5%/3 mm, TH = 10%) criteria using the best fraction as baseline. From the slope of the linear fit to the data (not shown for clarity) we obtained the change of every dosimetric parameter per 10% loss of passing rate

When using the best fraction as baseline, of the nine patients with failing fractions, six were patients with left‐sided breast cancer. Laterality is a determining factor in the study of the dosimetric parameters of the OARs, especially for the heart. Strong correlation for the lung parameters was found in four patients (patients 1, 4, 5, and 6) and moderate in another two patients (patients 3 and 18). In contrast, a moderate correlation was found for the mean heart dose in five patients and only in one patient a strong correlation was found (patient 4). The maximum heart dose was only correlated in three patients (moderately for patients 1 and 5, strong for patient 18). In the patients with correlation in the lung dosimetric parameters, a decrease in the absorbed dose in the lung was observed. Table 2 presents the change for the OARs dosimetric parameters from the nine patients with failing fractions using the best fraction as baseline. Figure 4 represents ΔV20Gy for the six patients with correlation between transit images passing rate and ipsilateral lung dose decrease. Differences between patients are observed: while for patient 3 or patient 1 there is always a decrease in the lung dose compared to the planned value (ΔV20Gy < 0). On the other hand, for patients 13 and 18, there are several fractions with passing rate values > 90% in which there is an over‐irradiation of the lung with respect to the planned value ΔV20Gy > 0. Same behavior is observed in the rest of the OARs dosimetric parameters studied.

**TABLE 2:** Change in OARs dosimetric parameters per every 10% decrease in passing rate with γ(5%/3 mm, TH = 10%) criteria using best fraction as baseline

		Change in dosimetric parameter per every 10% passing rate						
		Lung					Heart	
Patient	Laterality	ΔD_mean_	∆V5Gy	∆V10Gy	∆V17Gy	∆V20Gy	ΔD_mean_	ΔD_max_
1	Left	−1.7%	−2.9%	−2.7%	−2.2%	−2.1%	−0.3%	−10%
2	Right							
3	Left	−0.8%	−1.2%	−1.3%	−1.2%	−1.2%	−0.3%	
4	Left	−3.2%	−9.1%	−4.8%	−3.6%	−3.5%	−1.3%	
5	Left	−0.8%	−2.1%	−1.4%	−0.9%	−0.9%	−0.3%	−19%
6	Left							
13	Right	−1.5%	−3.3%	−3.4%	−2.2%	−2.0%	−0.1%	
14	Left						−0.4%	
18	Right	−1.8%	−3.1%	−3.0%	−2.8%	−2.5%		+0.1%

*Note*: Patients without correlation for a dosimetric parameter were excluded from the analysis (marked as grey in the table).

**FIGURE 5:**
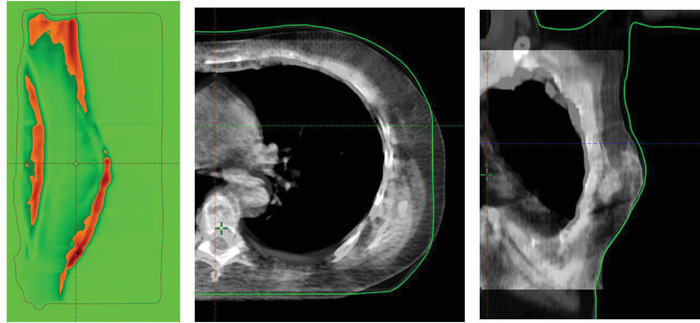
Eighth fraction of patient 3. Differences in passing rate are in the chest wall and the outer contour of the breast. CBCT images show differences in the same areas

**FIGURE 6:**
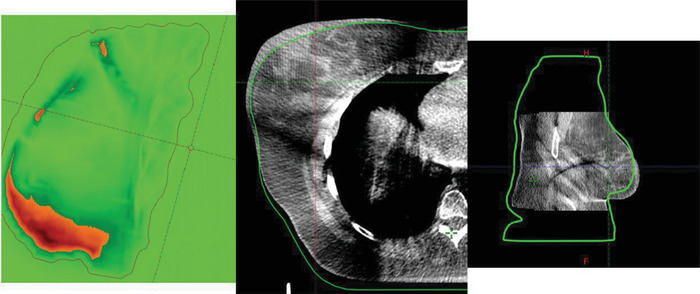
Thirteenth fraction of patient 2. Differences in passing rate are in the lower part of the breast. CBCT images show that the breast is out the external contour in this region, due to an inflammation process

In eight out of nine patients the causes of passing rate failure were related to errors during patient positioning. In patient number two the cause of failure was breast inflammation. In the first case, the areas with gamma values < 1 were in anatomical boundaries as the ribs or the scapula (Figure 5). In a swelling breast the areas with failing gamma criteria were in the breast while there were not differences in the anatomical boundaries (Figure 6).

## 4 DISCUSSION

We found an average change value of ‐11.6% [‐21.4%, ‐3.3%] and ‐3.2% [‐1.0%, ‐10.3%] for the PTVD98% and PTVD95% per every 10% decrease in the passing rate with γ(5%/3 mm, TH = 10%) criteria. Our results were obtained from the nine patients with failing fractions using the best fraction as a baseline. In the rest of the dosimetric parameters studied, no correlations were obtained in all patients with failing fractions. In the case of the parameters related to the maximum PTV dose, correlations were obtained in only four patients. We did not include the patients with no failing fractions as there was negative or no correlation between the change in dosimetric parameters and the gamma passing rate. The lack of correlation in patients without failing fractions is due to the fact that dosimetric parameters are near constant between fractions. As an example, in patient 7 all fractions had passing rates > 90% with γ(2%/2 mm, TH = 10%) and the maximum ∆D98% was 1%.

Differences in the change of dose coverage varied between patients. Thus, it is not feasible to predict accurately the variation of the dosimetric parameters based only on the passing rate of the transit image. The difference in the dose delivered depends not only on anatomical and positional changes but also on the robustness of the plan.^22^ As an example, a patient with a swelling breast, like patient 2, will reduce the signal of the transit image thus decreasing the passing rate, but if the photon fluence is extended outside the body contour using the skin flash tool this swollen part of the breast will indeed receive the planned dose. In this case the loss of coverage will be smaller than a poorly positioned patient, like patient 3 shown in Figure 5. However, the average change values of ‐11.6% and ‐3.2% for the PTVD98% and PTVD95% per every 10% passing rate decrease can be a helpful tool to estimate the dosimetric impact and determine when it is prioritary to take an action over the failing fractions.

Regarding the OARs, in six patients we observed a decrease in the ipsilateral lung dose. However, as Figure 4 shows, this behavior was not observed in all fractions for every patient. In some patients, there were fractions with an overdosage of the lung with respect to the planned values but there were not failing fractions. To study when a difference in positioning results in an increase or a decrease of the lung dose we use the Eclipse plan uncertainty parameters tool. We evaluated an isocenter shift of 0.5 cm in the set‐up of patient. We observed that a displacement of the isocenter of 0.5 cm out of the midline resulted in a decrease of the lung dose (∆V20Gy = ‐2.2%) and at the same time a loss of coverage in the PTV (ΔPTVD95% = ‐1%). Conversely, a shift towards the midline increased the lung dose (∆V20Gy = +2.2%) but will not affect the coverage as the PTV will enter the skin flash region. The same effect is magnified if the displacement of the isocenter is in the anterior direction; decrease of absorbed dose in the lung (∆V20Gy = ‐3.4%) and loss of PTV coverage (ΔPTVD95% = ‐2.4%). When the displacement is in the posterior direction the lung dose will increase more (∆V20Gy = +3.4%) without affecting PTV coverage. The displacements that have the least effect are those that occur in the caudal cranium direction. Although in clinical practice patients often experience displacements in all three axes, rotations and anatomical changes, this tool provides information on the dosimetric impact of differences in positioning. The fact that the analysis of several of our patients shows a decrease in lung dose implies that radiotherapy technicians in case of doubt have a tendency not to over‐irradiate the lung. Figure 5 shows this situation where the breast in the CBCT is posterior to the CT (the external contour of the CT is represented by the green line). This produces differences in the transit dosimetry in these areas of the breast and lung.

In this work, 13.9% of the analyzed transit images did not meet the tolerance level, established in a passing rate above 90% with γ(5%/3 mm, TH = 10%) criteria. Bossuyt et al.^23^ reported an 8% of failure images in breast patients with the same passing rate of acceptance but using a γ(7%/5 mm, TH = 20%) criteria with the PerFraction software. This is a higher number of failing fractions using a less restrictive criterion. Nevertheless, they present a bigger cohort of patients which could explain this difference. Also, failing fractions are related to patient immobilization, IGRT protocols, planning techniques, treatment protocols, etc., so it is not easy to compare between centers. Moreover, as the present study was retrospective, no actions could be taken. As an example, Halcyon patient 2 had a swelling breast with 12 failing fractions. This patient could probably be a candidate for replanning in a prospective study.

Fidanzio et al.^16^ reported an 8% of breast fractions failing with a tolerance of the quotient between measured and planned dose of 6% using EPID images to calculate dose at a reference point in the planning CT (half breast thickness along CAX). Nailon et al.^24^ obtained a 7.8% of failing fractions with the Dosimetry Check software. They applied a tolerance of 10% to the difference between calculated point dose and measure dose.

We only evaluated the breast PTV and excluded from analysis the boost PTV. Changes in boost volume during radiotherapy have been reported in the literature.^25^ However, if the boost migrates or changes its volume without altering the breast external contour significantly, the attenuation of the radiation beam will be the same. Therefore, transit dosimetry will not detect such changes. In these cases, it would be best to have an adaptive radiotherapy approach.^26^ Furthermore, all the patients analyzed were treated in free‐breathing mode, as at the time the study was made Halcyon did not allowed breathing control mechanisms. Hence, the results of this study would not be directly extrapolated if some type of respiratory control, such as deep inspiration breath‐hold (DIBH), were used.

In the study of correlations, the use of the first fraction as baseline yielded inconsistent results in correlations between the change in dosimetric parameters and the gamma passing rates. These inconsistencies can be explained by the fact that in three patients (5, 6, and 9) the first fraction ranked below the top ten fractions. In patient 6, despite having failing transit images, the correlation coefficient was negative. This means that when the lower the passing rate, and therefore the transit image is more different with the baseline, the lesser the change in coverage of the PTV. On the contrary, when using the best fraction as baseline, a positive correlation was found between passing rates of the transit images and the coverage of the PTV. Also, failing fractions of patients 3, 4, and 5 only were obtained when using best fraction fraction as baseline.

At the present time the only way to use transit images in clinical practice in Halcyon is to use the first fraction image as a baseline. The major drawback of this method is that it would not detect a deviation present in all the treatment fractions, such as an incorrect use of an immobilizer or a missing bolus. Even so, this work showed that using first fraction transit image as baseline produced suboptimal results, as it did not allow detection of all patients with failing fractions. However, in clinical practice it is not possible to know a priori which fraction will be delivered most similar to the treatment plan. The user could make an analysis of the first five fractions, as in 55% of the patients the best session was in these fractions. However, it is a difficult workload to implement in daily practice. Although it has not been analyzed in this study, we believe that one way to overcome this limitation is using a predicted dose image instead using a baseline image. In this case, every fraction will be compared with an image calculated from the treatment plan. Unfortunately, this is not possible in Eclipse. Even though commercial software as PerFraction includes an algorithm to calculate a predicted transit dose, it is not available for Halcyon linac at the present time. Therefore, currently the only way to use transit images in clinical practice in Halcyon is to use the first session as a baseline.

Although this has been shown to lead to non‐detection of failing fractions, the results of PTV coverage loss can help in decision making over failing images.

Besides that, the main difficulty we encounter in this work was determining the cause of a failure based in the transit image. Further research may include a study comparing the use of predicted dose image and baseline image as reference image in breast treatments, and to implement tools as deep learning^27^ to aid to classify the cause of errors.

## 5 CONCLUSIONS

We have studied the results of transit images of breast treatment plans treated in a Halcyon linac with the Portal Dosimetry tool using different gamma criteria. Using the transit images obtained from the inner tangential field we correlated the change in dosimetric PTV parameters with the decrease in gamma passing rate. The parameters that correlate best are those related to PTV coverage loss.

We found that using a baseline image can lead to the non‐detection of failing fractions. However, as this is currently the only way to use the transit images in Halcyon average values of PTV coverage loss can help user who decide to implement transit dosimetry in Halcyon.

## AUTHOR CONTRIBUTIONS

All authors contributed to the conception of the study, data analysis and manuscript writing. Data collection: David Sánchez‐Artuñedo, Victoria Reyes López, Raquel Granado Carrasco and Marcelino Hermida‐López.

## CONFLICT OF INTEREST

The authors declare no conflict of interest
